# Predicting postoperative coronal alignment after fixed-bearing unicompartmental knee arthroplasty using a new morphological assessment method: the arithmetic hip-knee-ankle angle

**DOI:** 10.1007/s00264-023-06072-6

**Published:** 2023-12-27

**Authors:** Naoki Nakano, Masanori Tsubosaka, Tomoyuki Kamenaga, Yuichi Kuroda, Kazunari Ishida, Shinya Hayashi, Ryosuke Kuroda, Tomoyuki Matsumoto

**Affiliations:** 1 Department of Orthopaedic Surgery, Kobe University Graduate School of Medicine, 7-5-1 Kusunoki-Cho, Chuo-Ku, Kobe, Hyogo 650-0017 Japan; 2 Department of Orthopaedic Surgery, Kobe Kaisei Hospital, 3-11-15, Shinoharakita-Machi, Nada-Ku, Kobe, Hyogo 657-0068 Japan

**Keywords:** Unicompartmental knee arthroplasty, Coronal alignment, Arithmetic hip-knee-ankle angle, Lateral distal femoral angle, Medial proximal tibial angle

## Abstract

**Purpose:**

Only a few reports have been published so far on factors that predict postoperative coronal alignment after unicompartmental knee arthroplasty (UKA). The purpose of this study is to clarify the relationship between the arithmetic hip-knee-ankle angle (aHKA) and postoperative coronal alignment after medial fixed-bearing UKA.

**Methods:**

One hundred and one consecutive patients (125 knees) who underwent medial fixed-bearing UKA were assessed. Pre- and postoperative coronal HKA angles, lateral distal femoral angle (LDFA), medial proximal tibial angle (MPTA), and the thickness of the tibial and femoral bone cut were measured. aHKA was calculated as 180° − LDFA + MPTA. Correlations between postoperative HKA angle and aHKA, LDFA, and MPTA were investigated by single regression analysis. After the patients were divided into three groups according to the postoperative HKA angle, i.e., HKA angle > 180°, 175° < HKA angle ≤ 180°, and HKA angle ≤ 175°, aHKA, LDFA, MPTA, preoperative HKA angle, and the thickness of the distal femoral as well as tibial bone cut were compared among the three groups.

**Results:**

aHKA and MPTA were positively correlated with postoperative HKA angle, while no correlation was found between postoperative HKA angle and LDFA. Among the three groups classified by postoperative HKA angle, significant differences were found in aHKA, MPTA, and preoperative HKA angle, while no significant difference was found in LDFA and the amount of distal femoral and tibial osteotomies.

**Conclusions:**

aHKA was correlated with postoperative HKA angle after medial fixed-bearing UKA, which was probably due to the influence of MPTA.

## Introduction

Medial unicompartmental knee arthroplasty (UKA) is an effective treatment for isolated medial osteoarthritis (OA) or osteonecrosis (ON) of the medial femoral condyle [1], yielding successful functional outcomes, high satisfaction rates, and a fast recovery after surgery [2–7]. Also, it has been suggested that patients undergoing UKA have fewer complications than patients who underwent total knee arthroplasty (TKA) [8, 9], and it has previously been reported that UKA is a more suitable technique for Asian patients than TKA, as their lifestyle requires deep knee flexion [10].

However, some past studies have reported higher revision risk in UKA compared to TKA [11–14], and malalignment of the lower limb alignment has been identified as the major contributing factor to high revision rate, poor clinical outcomes, and early polyethylene wear [15, 16]. Mild under-correction is considered to be acceptable, while a consequence of excessive residual varus alignment is increased compartment force by overloading medially, which can lead to failure due to polyethylene wear [16–19]. On the contrary, overcorrection of the coronal alignment may result in degeneration of the lateral compartment and lead to premature loosening of the prosthesis [17, 18]. Therefore, a reproducible method of predicting postoperative coronal alignment is desired to assume the risk of postoperative coronal malalignment. However, only a few studies have reported the risk factors of postoperative coronal malalignment in UKA [20, 21].

Recently, a new morphological assessment method, the arithmetic hip-knee-ankle angle (aHKA), has attracted attention as a method of assessing lower limb morphology while the mechanical hip-knee-ankle angle has been used to assess coronal alignment traditionally [22]. To calculate aHKA, lateral distal femoral angle (LDFA) and medial proximal tibial angle (MPTA) are measured separately using long-leg radiographs, and an arithmetic method is subsequently used. aHKA is predictive of the patient’s constitutional knee alignment when comparing arthritic to non-arthritic patients [22]. Because aHKA uses only bony landmarks and is independent of the relationship of the femur to the tibia, it is not affected by joint space narrowing. At the same time, aHKA is not affected by whether the patient is standing or lying down at the time of imaging, so it is expected to have fewer measurement errors between patients.

The purpose of this study is to clarify the relationship between aHKA and postoperative coronal alignment after medial fixed-bearing UKA. We hypothesized that preoperative aHKA is correlated with postoperative HKA angle after medial fixed-bearing UKA.

## Materials and methods

The hospital ethics committee approved the study protocol (No. 1510, Date of Approval: 02 Dec 2013) of this study. One hundred and one consecutive patients (125 knees) underwent medial fixed-bearing UKA using the Persona Partial Knee System (Zimmer Biomet Inc., Warsaw, IN, USA) and informed consent was obtained from all the patients, and the obtained data were retrospectively collected and analyzed. The inclusion criteria for UKA were a radiographic diagnosis of isolated end-stage (Kellgren-Lawrence grade 4) medial compartment OA or ON with an active ROM of ≥ 90°, a fixed flexion deformity of ≤ 15°, and a varus deformity of ≤ 10°. Of ON, those with end-stage OA in the background were treated as OA. Magnetic resonance imaging was performed for all the cases before surgery and the cases without the intact anterior cruciate ligament and articular surface of the lateral compartment were treated with TKA, not UKA. All the 125 UKAs were included in the study. Senior two surgeons with > 15 years of experience performed the surgery.

**Fig. 1:**
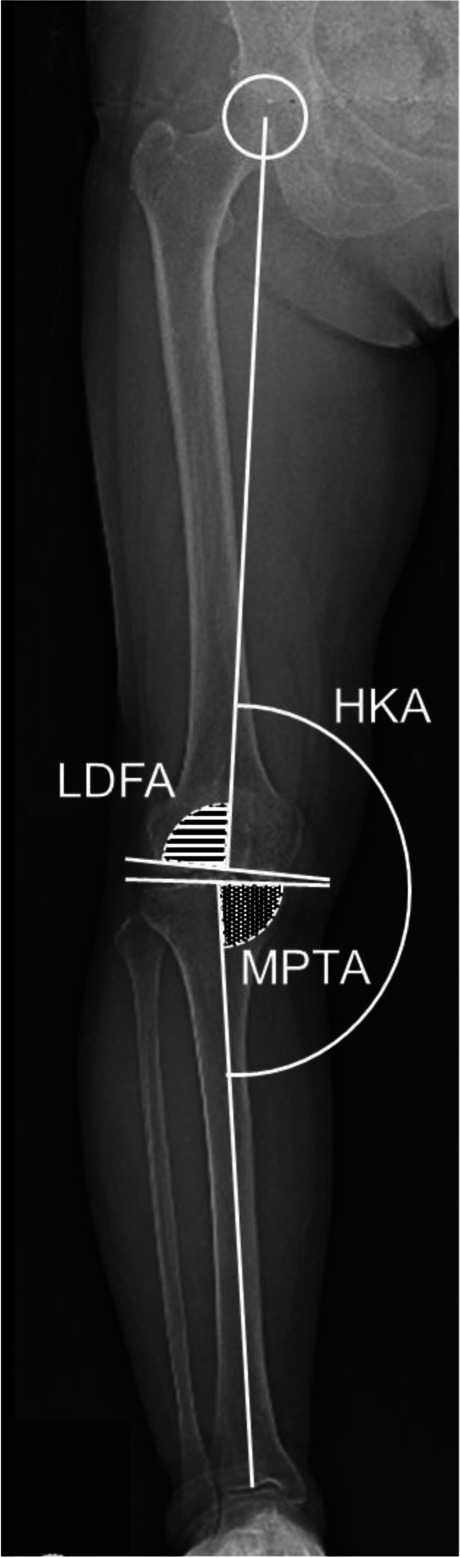
Measurement of different alignment parameters on the weight-bearing long-leg radiograph. HKA, hip-knee-ankle angle; LDFA, lateral distal femoral angle; MPTA, medial proximal tibial angle

**Fig. 2:**
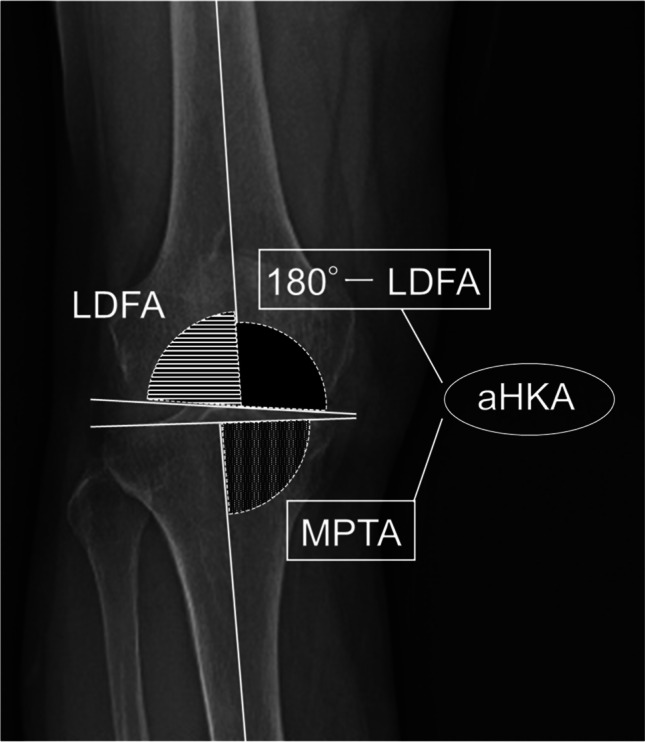
Measurement of the arithmetic HKA (aHKA). aHKA was calculated as 180° − LDFA + MPTA

**Fig. 3:**
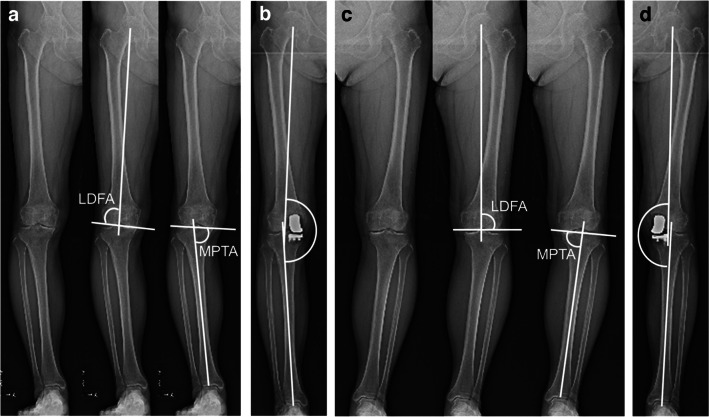
Preoperative long-leg standing radiographs in two cases to illustrate the calculation of aHKA. **a** Case 1: LDFA is 88.1° and MPTA is 83.3°. aHKA is calculated as 180° − LDFA (°) + MPTA (°) = 175.2°. **b** Case 1: postoperative HKA angle is 175.7°. **c** Case 2: LDFA is 89.2° and MPTA is 87.4°. aHKA is calculated as 180° − LDFA (°) + MPTA (°) = 178.2°. **d** Case 2: postoperative HKA angle is 178.4°

After inflating the tourniquet to 250 mmHg, a limited-medial parapatellar approach was performed. Following the macroscopic observation of an intact articular surface of the lateral as well as patellofemoral compartments, the minimal soft tissue release of the medial structures and osteophyte removal were performed. A proximal tibial osteotomy was then performed using an accelerometer-based portable navigation system (OrthAlign Plus®, UniAlign™; OrthAlign Inc., Aliso Viejo, CA, USA), which helps the surgeon to precisely perform tibial bone cut in UKA with coronal and sagittal alignment. It provides a measurement accuracy of ± 0.5° when measuring the angle between the OrthAlign Plus® unit and the reference sensor (manufacturer’s data). The target value of preoperative planning in coronal alignment was set to neutral (0°) in varus. Sagittal alignment was set regarding the original posterior tibial slope, which was measured with reference to the perpendicular line of the sagittal axis. The tibial sagittal axis was defined as the line connecting the anterior one-third of the medial tibial plateau and midpoints of the tibial plafond. The sagittal alignment was set to 6.0° and 8.0° when the angle was ≤ 6.0° and ≥ 8.0°, respectively, and the target value was set as the target value of preoperative planning when the angle was between 6.0° and 8.0° as in previous studies [23, 24]. The target osteotomy volume of the tibia (excluding the thickness of the bone saw blade) was set to 4 mm using a special stylus in all the cases. Following the tibial osteotomy, a distal femoral osteotomy was performed with the spacer block technique referring to the surface of the proximal tibial cut. The femoral rotation was carefully adjusted to the mechanical axis of the tibia, and the remaining (posterior and chamfer part) osteotomies of the femur were performed. The thickness of the tibial and femoral bone cut was measured using a calliper and the actual osteotomies were calculated by adding the thickness of the bone saw blades (1.27 mm). Pre- and postoperative coronal HKA angles, LDFA, and MPTA were measured based on long-leg standing radiographs (Fig. 1). LDFA was measured as the lateral angle formed by the mechanical axis of the femur and a line drawn across the articular surface of the distal femur at the most distal points of the lateral and medial femoral condyles. MPTA was measured as the angle formed medially by the mechanical axis of the tibia and a line drawn between the most distal articular contours of the midpoints of the lateral and medial plateaus. aHKA was calculated as 180° − LDFA + MPTA (Fig. 2). Two examples of actual aHKA calculation are given to advance understanding using preoperative long-leg standing radiographs. Case 1 (Fig. 3a, 3b): LDFA is 88.1° and MPTA is 83.3°. aHKA is calculated as 180° − LDFA (°) + MPTA (°) = 175.2°. Postoperative HKA angle is 175.7°. Case 2 (Fig. 3c, 3d): LDFA is 89.2° and MPTA is 87.4°. aHKA is calculated as 180° − LDFA (°) + MPTA (°) = 178.2°. Postoperative HKA angle is 178.4°.

The data are expressed as means ± standard deviations. To determine the intra-observer and inter-observer reliability of the measurements of LDFA, MPTA, and HKA angle, two investigators assessed the first ten patients twice and calculated intra-class correlation coefficients (ICCs). The ICCs for intra-observer reliability were > 0.84 (range 0.84–1.00) and those for inter-observer reliability were > 0.82 (range 0.82–0.96) for all measurements. Correlations between postoperative HKA angle and aHKA, LDFA, and MPTA were investigated by single regression analysis. Also, patients were divided into three groups according to the postoperative HKA angle, i.e., group valgus (HKA angle > 180°), group mild varus (175° < HKA angle ≤ 180°), and group severe varus (HKA angle ≤ 175°). The Tukey–Kramer test was utilized to compare aHKA, LDFA, MPTA, preoperative HKA angle, the thickness of the distal femoral bone cut, and the thickness of the tibial bone cut among the three groups. Data analyses were performed using BellCurve for Excel (Social Survey Research Information Co., Ltd., Tokyo, Japan). The sample size calculation was performed using G*Power 3 (Heinrich Heine Universität Düsseldorf, Germany). Based on our calculations, a minimum sample size of 89 patients was required for the single regression analysis with an effect size *f*^2^ of 0.15, a type I error (*α*) of 0.05, and a power (1 − *β*) of 0.95. Statistical significance was considered at *P* < 0.05.

## Results

A total of 125 knees in 101 patients were enrolled in the study. The mean ± SD of age was 74.04 ± 8.1 years (range 51–89 years). Of all cases, 39 (31.2%) were male, and 86 (68.8%) were female; 59 (47.2%) had surgery on the left knee, and the others (66 (52.8%) were on the right knee; 106 (84.8%) were diagnosed as OA, and 19 (15.2%) were diagnosed as ON (Table 1). The mean ± SD of radiological parameters (including preoperative and postoperative HKA angle, LDFA, MPTA, and aHKA), the amount of distal femoral osteotomy, and the amount of tibial osteotomy are presented in Table 1. Of the 125 knees included in our study, 24 knees, 77 knees, and 24 knees were classified as group valgus, group mild varus, and group severe varus, respectively.

**Table 1:** Basic characteristics

Variables		
Age (years)		74.04 (range 51–89; SD 8.1)
Gender (male: female)		30 (39 UKAs): 71 (86 UKAs)
Side of UKA (left: right)		59: 66
Diagnosis (OA: ON)		106: 19
aHKA (°)		177.2 (range 172.3–182.4; SD 2.1)
LDFA (°)		87.8 (range 84.1–90.4; SD 1.2)
MPTA (°)		85.0 (range 81.1–89.1; SD 1.9)
Preoperative HKA angle (°)		172.4 (range 163.8–179.1; SD 2.8)
Postoperative HKA angle (°)		177.0 (range 170.2–183.0; SD 2.5)
Amount of distal femoral osteotomy (mm)		6.3 (range 4.3–9.3; SD 0.8)
Amount of tibial osteotomy (mm)		5.1 (range 3.3–7.3; SD 0.9)

The values are given as the mean and standard deviation for continuous variables

*UKA*, unicompartmental knee arthroplasty; *OA*, osteoarthritis; *ON*, osteonecrosis; *HKA*, hip-knee-ankle; *SD*, standard deviation; *aHKA*, arithmetic hip-knee-ankle angle; *LDFA*, lateral distal femoral angle; *MPTA*, medial proximal tibial angle

**Fig. 4:**
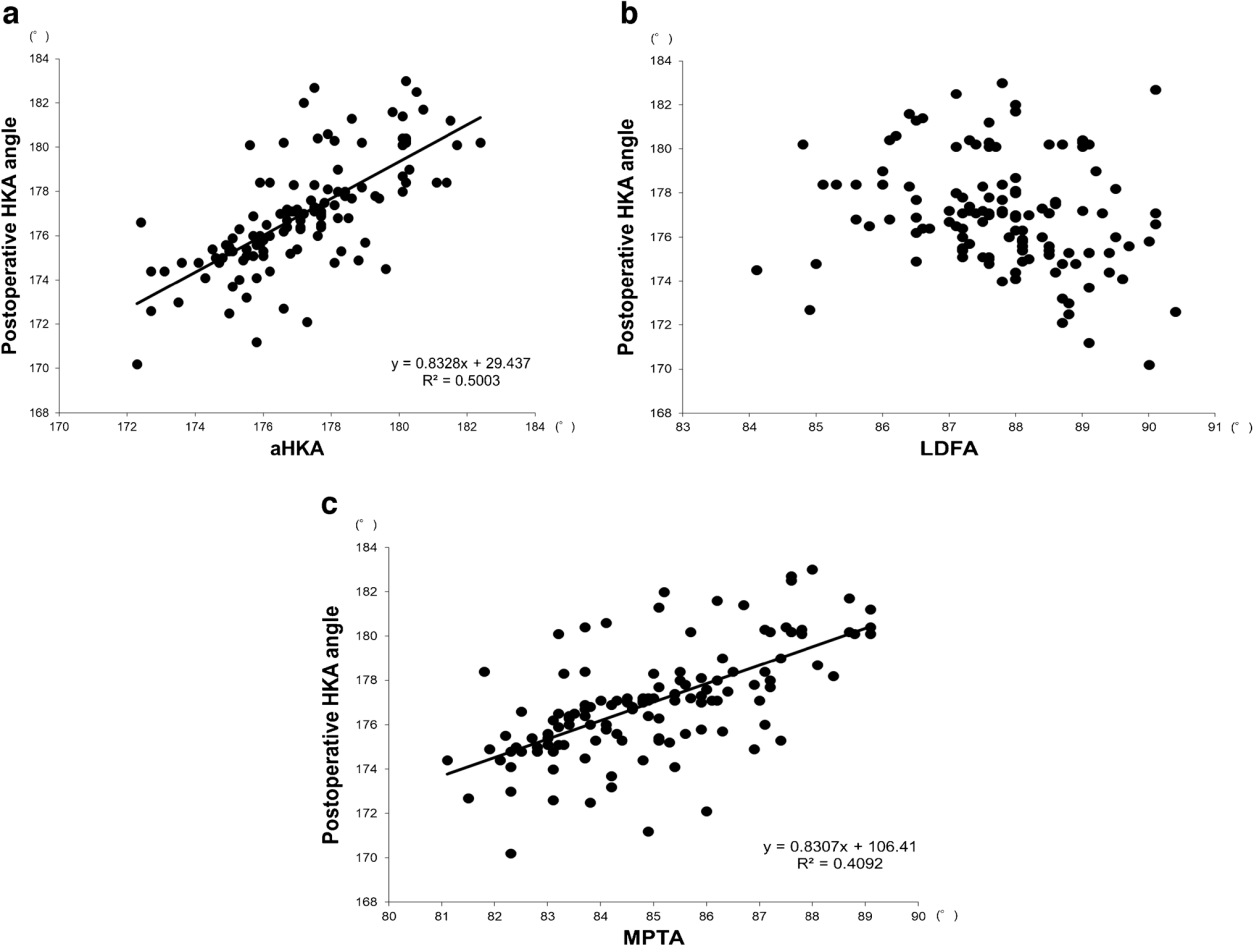
**a** Correlation analysis between aHKA and postoperative HKA angle. The aHKA was positively correlated with the postoperative HKA angle. **b** Correlation analysis between LDFA and postoperative HKA angle. No correlation was found between the two measurements. **c** Correlation analysis between MPTA and postoperative HKA angle. MPTA was positively correlated with the postoperative HKA angle

**Fig. 5:**
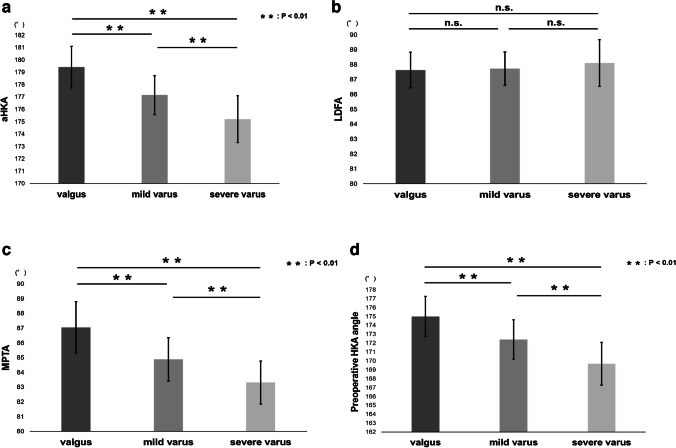
**a** Mean and the standard deviation of aHKA for each postoperative HKA angle group. Significant differences were found between each of the three groups (*P* < 0.01). **b** Mean and the standard deviation of LDFA for each postoperative HKA angle group. No significant difference was found among the three groups. **c** Mean and the standard deviation of MPTA for each postoperative HKA angle group. Significant differences were found between each of the three groups (*P* < 0.01). **d** Mean and the standard deviation of the preoperative HKA angle for each postoperative HKA angle group. Significant differences were found between each of the three groups (*P* < 0.01)

**Fig. 6:**
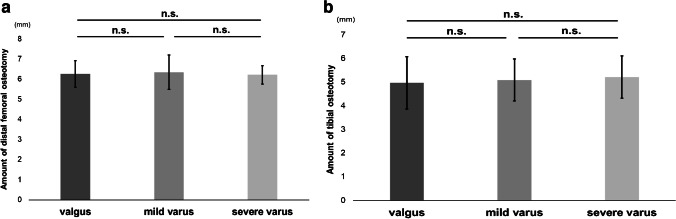
**a** Mean and the standard deviation of the amount of distal femoral osteotomy for each postoperative HKA angle group. No significant difference was found among the three groups. **b** Mean and the standard deviation of the amount of tibial osteotomy for each postoperative HKA angle group. No significant difference was found among the three groups

As for the correlation analyses between postoperative HKA angle and aHKA, LDFA, or MPTA, aHKA (*R*^2^ = 0.5003) and MPTA (*R*^2^ = 0.4092) were positively correlated with postoperative HKA angle (*P* < 0.001), while no correlation was found between postoperative HKA angle and LDFA (Fig. 4). In comparisons between the three groups classified according to the postoperative HKA angle, i.e., group valgus, group mild varus, and group severe varus, significant differences were found among the three groups in aHKA, MPTA, and preoperative HKA angle, while no significant difference was found among the three groups in LDFA (Fig. 5), which were consistent with the results from the correlation analyses. Regarding the amount of distal femoral osteotomy, no significant difference was found among the three groups, and the same was true with regard to the amount of tibial osteotomy (Fig. 6).

## Discussion

The main finding of this study was that the aHKA was positively correlated with postoperative HKA angle (*R*^2^ = 0.5003) (Fig. 4a), and analysis of each element of aHKA showed that MPTA was positively correlated with postoperative HKA angle (*R*^2^ = 0.4092) (Fig. 4c) while no correlation was found between LDFA and postoperative HKA angle (Fig. 4b). Similar results were obtained when the cases were compared by dividing them into three groups by postoperative HKA angle (Fig. 5a–c). Overall, the preliminary hypothesis was proved to be correct. Specific examples show that case 1 (Fig. 3a; 175.2°) has aHKA smaller than case 2 (Fig. 3c; 178.2°), and case 1 (Fig. 3b; 175.7°) is also smaller than case 2 (Fig. 3d; 178.4°) in terms of postoperative HKA angle, which is in line with the trend shown in this study.

Also, many of the cases were end-stage medial compartment OA (Kellgren-Lawrence grade 4) and the medial joint space was considered to have almost disappeared, which was assumed to be a factor in the significant association between pre- and postoperative HKA angle (Fig. 5d). Also, just to be sure, the amount of osteotomy in each group was compared, but there was no significant difference, indicating that the present results were not due to the effect of the amount of osteotomy (Fig. 6). The current study first explored the relationship between aHKA and postoperative HKA in fixed-bearing UKA.

UKA is essentially only a resurfacing procedure and the osteotomy angle does not cause alignment changes. Given the lack of influence of the osteotomy volume on the results, the original bone morphology, i.e., aHKA, is considered to be directly reflected in the postoperative HKA angle. Liu et al. showed that aHKA was correlated with the HKA angle after mobile-bearing UKA and a high aHKA (> 180°) would increase the risk of postoperative valgus malalignment. They concluded that in patients with preoperative aHKA > 180°, mobile-bearing UKA should be conducted with caution [25]. The present study showed the same to be true for fixed-bearing UKA with the spacer block technique, and in addition, aHKA was shown to be a useful indicator of the risk of excessive postoperative varus malalignment.

aHKA has attracted attention in recent years as a method of assessing bone morphology in the lower limb with the rising interest in kinematic techniques in TKA, which aims to restore pre-arthritic alignment [26], as it is based on a practical, straightforward method for estimating constitutional lower limb alignment after the development of arthritic deformity. LDFA and MPTA do not change in the process of joint space narrowing in the absence of significant bone loss. In other words, the calculation of the aHKA is not affected by joint space narrowing or tibiofemoral subluxation and is independent of the joint line convergence angle, which has been shown in a past study [27]. In this study, HKA angle, LDFA, and MPTA were measured using long-leg standing radiographs, which have been reported to show a good correlation with those measured by CT, and the use of long-leg standing radiographs was considered to be acceptable because of its simplicity [28]. In UKA, aHKA has recently been used to assess lower limb alignment. Plancher et al. investigated if pre-arthritic/kinematic alignment of knees results in sustained long-term restoration of function, without conversion to TKA, following 150 fixed-bearing medial UKAs at an average of ten years after surgery. As a result, patients with compared to those without pre-arthritic/kinematically aligned knees had significantly longer mean survival and higher KOOS Activities of Daily Living and Sport subscale scores [29]. Bayoumi et al. assessed whether patients with pre-arthritically aligned knees demonstrated better mid-term outcomes and survivorship compared to patients with non-pre-arthritically aligned knees following medial fixed-bearing UKA using aHKA algorithm. As a result, pre-arthritically aligned knees and knees with relative overcorrection from their pre-arthritic alignment following UKA demonstrated better mid-term outcomes and survivorship than knees with relative under-correction from their pre-arthritic alignment [30].

Although UKA is a successful procedure for medial OA and ON, failures still exist in patients undergoing UKA, and lateral OA progression is one of the major complications that lead to conversion to TKA [31, 32]. Valgus malalignment, i.e., overcorrection of the lower limb alignment following UKA, is one of the most common reasons for the progression of lateral OA [33, 34]. For example, a past study found that patients with progressive lateral OA had valgus malalignment after UKA in a cohort of 3351 knees with a minimum follow-up of ten years [34]. It is therefore very important to find out the risk factors of postoperative valgus malalignment and proactively avoid them. Conversely, problems have also been reported for varus malalignment after UKA. Slaven et al. reported that when 61 revision cases for loosening after medial fixed-bearing UKAs were compared with 61 matched clinical success UKAs, the loosening group showed the mean HKA angle was 6.1° ± 3.1° of varus while the matched success group showed the mean HKA angle was 4.0° ± 2.7° of varus (*P* < 0.001) [35]. Also, Foissey et al. described patients with joint line lowering and postoperative varus malalignment were at high risk of tibial implant failure in 366 UKAs with a mean follow-up period of 61.3 months [36]. Given these, in medial fixed-bearing UKA with the use of the spacer block technique referring to the surface of the proximal tibial cut, it may be necessary to increase or decrease the amount of tibial osteotomy depending on the preoperative aHKA and consider the thickness of the insert used to maintain proper postoperative alignment.

This study had some limitations. Firstly, we assessed the relationship between the postoperative HKA angle and a new morphological indicator (aHKA) while we did not investigate the impact of lower-leg alignment after UKA. Therefore, further long-term follow-up of patients is required to clarify the relationship between postoperative alignment and clinical outcomes including patient-reported outcome measurements. Secondly, radiographic measurements, including pre- and postoperative HKA angle, LDFA, and MPTA, were still affected by the rotation of lower extremity and osteophyte formation while both the intra-observer and inter-observer agreements for these measurements were well within acceptable limits. Lastly, this study was a retrospective analysis involving fixed-bearing medial UKA with the spacer block technique only. Therefore, it is unclear if the current results are applicable for the mobile-bearing medial UKA or the fixed-bearing medial UKA with the use of other surgical techniques, e.g., the intramedullary rod method.

## Conclusions

aHKA was correlated with postoperative HKA angle after medial fixed-bearing UKA, and it was assumed to be mainly due to the influence of MPTA. aHKA, an indicator of bony nature unaffected by loading or joint space narrowing, was considered useful in predicting the occurrence of postoperative coronal malalignment after UKA.

## Data Availability

Authors can confirm that all relevant data are included in the article.
